# Advances in Biosynthesis of Non-Canonical Amino Acids (ncAAs) and the Methods of ncAAs Incorporation into Proteins

**DOI:** 10.3390/molecules28186745

**Published:** 2023-09-21

**Authors:** Liang Chen, Xiulan Xin, Yuning Zhang, Shunyao Li, Xiaoman Zhao, Song Li, Zhaochu Xu

**Affiliations:** College of Bioengineering, Beijing Polytechnic, Beijing 100176, China; xinxiulan@bpi.edu.cn (X.X.); zhangyuning@bpi.edu.cn (Y.Z.); lishunyao@bpi.edu.cn (S.L.); zhaoxiaoman@bpi.edu.cn (X.Z.); lisong@bpi.edu.cn (S.L.); xuzhaochu@bpi.edu.cn (Z.X.)

**Keywords:** non-canonical amino acid, genetic code expansion, biosynthesis, incorporation, tailor-made protein

## Abstract

The functional pool of canonical amino acids (cAAs) has been enriched through the emergence of non-canonical amino acids (ncAAs). NcAAs play a crucial role in the production of various pharmaceuticals. The biosynthesis of ncAAs has emerged as an alternative to traditional chemical synthesis due to its environmental friendliness and high efficiency. The breakthrough genetic code expansion (GCE) technique developed in recent years has allowed the incorporation of ncAAs into target proteins, giving them special functions and biological activities. The biosynthesis of ncAAs and their incorporation into target proteins within a single microbe has become an enticing application of such molecules. Based on that, in this study, we first review the biosynthesis methods for ncAAs and analyze the difficulties related to biosynthesis. We then summarize the GCE methods and analyze their advantages and disadvantages. Further, we review the application progress of ncAAs and anticipate the challenges and future development directions of ncAAs.

## 1. Introduction

Proteins are essential to life processes, playing important roles in transport, catalysis, and regulation. Most proteins are composed of only 20 canonical amino acids (cAAs). While the encoding of cAAs is sufficient for fundamental growth and metabolic functions, proteins require additional chemical groups, such as ketone, aldehyde, azide, amide, nitro, and sulfonate, to perform more complicated and diverse biological functions [1,2,3]. These extra functional groups are necessary because the 20 cAAs severely limit the types and functional applications of proteins, which are no longer sufficient to meet the research needs of fields such as biological science, chemistry, and medicine [4,5].

Non-canonical amino acids (ncAAs) are derivatives of cAAs, also known as non-standard amino acids (nsAAs), unnatural amino acids (unAAs or uAAs), or non-proteinogenic amino acids (npAAs). Some examples of ncAAs include L-homoserine, which serves as a precursor for the synthesis of essential amino acids and other chemical products [6,7]. 5-Aminolevulinic acid is an important precursor for the biosynthesis of heme, porphyrin, chlorophyll, and vitamin B12 [8]. NcAAs contain various functional groups, such as ketyl, alkynyl, azide, nitro, phosphate, and sulfonate, which enable them to modify proteins. So far, more than 200 ncAAs can be inserted proteins using genetic code expansion (GCE) technologies [9,10], which are useful for the research on protein structures and functions.

Despite the growing diversity and range of applications of ncAAs, their synthesis is still a challenge [11,12,13]. The traditional chemical synthesis methods are limited by harsh reaction conditions [14], highly volatile toxic substances [15,16], environmental pollution and high raw material costs [17]. Metabolic engineering is an emerging technique that could potentially solve these issues and enable the green and efficient production of ncAAs. Based on that, this study reviews the methods, applications, difficulties, and solutions of metabolic engineering biosynthesis of ncAAs. The methods of incorporating ncAAs into proteins using GCE methods are also summarized. The potential applications of tailor-made proteins are also discussed, and the future applications are predicted.

## 2. Biosynthesis of ncAAs

As previously mentioned, the chemical synthesis of ncAAs has significant drawbacks. In addition, ncAAs have high technical barriers to synthesis due to the limitations of key technologies such as the screening and preparation of chemical catalysts, the construction of process routes, and the regulation of catalytic processes [18]. The development of a green and efficient synthesis method for ncAAs is crucial. Metabolic engineering offers promising solutions for synthesizing ncAAs by understanding the catalytic mechanisms of related enzymes. The key heterologous enzymes can be recombined, modified, and optimized in the engineered microbes. These microbes create the possibility of establishing a production platform for ncAAs. The usage of metabolic engineering offers a promising path toward creating a sustainable and cost-effective method for producing ncAAs.

### 2.1. 5-Hydroxytryptophan

**Figure 1:**
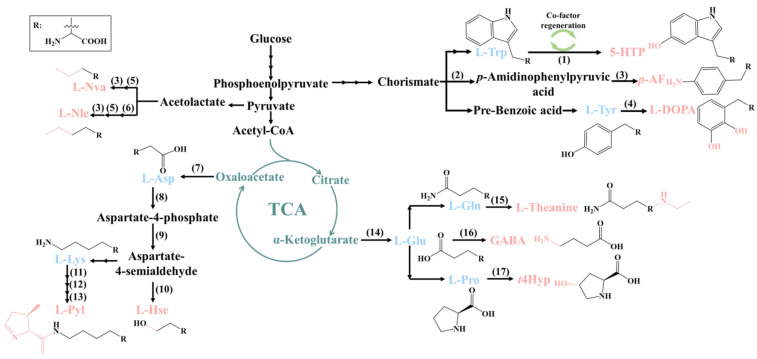
Biosynthetic pathways of some ncAAs derived from glucose. The blue color indicates the cAAs. The pink color indicates the ncAAs. Some key enzymes in the pathways: (1) Tryptophan hydroxylase or aromatic amino acid hydroxylase; (2) PapA, PapB and PapC; (3) Branched-chain amino acid transaminase; (4) Tyrosine phenol-lyase; (5) LeuABCD; (6) IlvCD; (7) Aspartate amino transferase; (8) Aspartokinase; (9) Aspartate-semialdehyde dehydrogenase; (10) Homoserine dehydrogenase; (11) Radical SAM enzyme PylB; (12) ATP-dependent PylC; (13) PylD for oxidation; (14) Glutamate dehydrogenase; (15) Theanine synthetase; (16) Glutamate decarboxylase; (17) Proline-4-hydroxylase (P4H). Abbreviation: 5-HTP: 5-Hydroxy tryptophan; *p*-AF: *p*-amino-phenylalanine; L-DOPA: Levodopa (3,4-dihydroxy-L-phenylalanine); L-Nva: L-Norvaline; L-Nle: L-Norleucine; L-Pyl: L-Pyrrolysine; L-Hse: L-Homoserine; GABA: Gamma-aminobutyric acid; *t4*Hyp: *Trans*-4-hydroxyproline.

5-Hydroxytryptophan (5-HTP) is a compound with medicinal value that can be used to treat depression, insomnia, and other diseases. Wang et al. have successfully constructed a recombinant strain to biosynthesize 5-HTP [19]. The biosynthesis pathway was constructed on two plasmids containing three functional modules, namely substrate L-Trp biosynthesis modules, hydroxylation module, and cofactor regeneration module (Figure 1). Moreover, the human tryptophan hydroxylase I (TPH I) was introduced into the *E. coli* BL21Δ*tnaA* strain to hydroxylate L-Trp to produce 5-HTP. By further inserting the tryptophan synthesis pathway into the genome, the yield of 5-HTP in shake-flask fermentation was increased to 1.61 g/L while reducing the accumulation of precursor L-Trp, which was beneficial for the subsequent separation and purification of 5-HTP [20]. After that, Lin et. al engineered the phenylalanine-4-hydroxylase from *Xanthomonas campestris* (*Xc*P4H) and introduced it into a L-Trp-producing *E. coli* strain via a co-factor regeneration pathway. The engineered strain produced 1.2 g/L 5-HTP [21]. Mora-Villalobos utilized sequence analysis, phylogenetic analysis, and functional differential analysis tools to predict, screen, and design the specific mutations of substrate-specific sites of aromatic amino acid hydroxylase from *Cupriavidus taiwanensis* (*Ct*AAAH). The substrate preference of the *Ct*AAAH was transferred from L-Phe to L-Trp, enabling the generation of 5-HTP with L-Trp as substrate [22,23]. These studies indicate that microbial-based metabolic engineering has achieved the production of 5-HTP in a green, efficient, and low-cost manner.

### 2.2. L-Homoserine

L-Homoserine (L-Hse), also known as 2-amino-4-hydroxybutyric acid, is a valuable platform chemical that has been widely used in various fields, such as medicine, agriculture, cosmetics, and spices. The microbial fermentation method has great potential for the large-scale production of L-Hse. Recent studies have focused on using *E. coli* and *Corynebacterium glutamate* (*C. glutamate*) to achieve high-level production of L-Hse [24,25,26,27]. For example, Cai et al. enhanced the production of L-Hse using a non-auxotrophic deficient and plasmid-free *E. coli* chassis [28]. They first constructed a *E. coli* chassis host strain via the knock-down of the L-Hse degradation pathway [29]. Then, they optimized the metabolic flux of L-Hse biosynthesis by overexpressing the *ppc*, *aspC*, *aspA*, *thrA*^fbr^, and *lysC*^fbr^cgl (Figure 1). Additionally, they promoted L-Hse efflux by modifying the transport system, introduced a strategy of synergistic utilization of co-factors to promote the regeneration of NADPH, and coordinated the level of redox co-factors by incorporating a heterologous dehydrogenase. As a result, the engineered *E. coli* strain was able to produce 85.29 g/L of L-Hse in a 5-liter fermenter, which was the highest titer of the plasmid-free and non-auxotrophic strains reported to date. This study demonstrates the effectiveness of optimizing L-Hse production using metabolic engineering strategies. Although *E. coli* as an amino acid-producing chassis has achieved high-level production of L-Hse, large amounts of by-products such as acetate are also produced during the fermentation process [24,30]. Moreover, *C. glutamate*, which is known for its ability to synthesize useful compounds using cheap feedstock, was used to produce L-Hse. Through overexpressing key kinase genes, disrupting competing and degrading pathways, and promoting the synthetic flux, the engineered *C. glutamate* produced 8.8 g/L L-Hse in a shake flask [26].

### 2.3. Trans-4-Hydroxyproline

*Trans*-4-hydroxyproline (*t*4Hyp) is a value-added amino acid that has been widely used in medicine, food, and cosmetics, especially in the field of chiral synthetic materials. *t*4Hyp is traditionally produced via the acidic hydrolysis of collagen, but the process has some drawbacks, such as low productivity and a complicated process. Metabolic engineering has been used to efficiently construct microbial cell factories of *E. coli* or *C. glutamate* in order to biosynthesize *t*4Hyp [31,32,33]. The introduction of a heterologous proline 4-hydroxylase from *Alteromonas mediterranea* (*Al*P_4_H) into *E. coli* enabled the accumulation of 45.83 g/L *t*4Hyp within 36 h in a 5-liter fermenter without the addition of proline [34]. The knockout of the genes of *putA*, *proP*, *putP*, and *aceA* in competing pathways and mutations of ProB to D107N/E143A ProB in order to alleviate the feedback inhibition of L-Pro maximized the production of L-Pro so as to enhance the biosynthesis of *t*4Hyp. Subsequently, the enzyme activity of L-Pro hydroxylase was increased using genome mining technology and rational design. Ultimately, the engineered strain produced 54.8 g/L *t*4Hyp in 60 h using glycerol and glucose as carbon sources [33]. The microbial metabolic network is large and complex. Genome modification methods such as gene knockout may lead to slow cell growth, stagnation, or even death, which may not be suitable for blocking some competing pathways. So far, CRISPR interference (CRISPRi) that can reduce the transcription of target genes by up to 1000-fold inhibition without miss effect [35,36] appeared as an alternative way to downregulate the expression of enzymes, which may be employed to repress the expression of the *putA* gene to further increase *t*4Hyp production in the future.

### 2.4. Other ncAAs

L-Pyrrolysine (L-Pyl) is the 22nd amino acid that has so far been discovered to insert into proteins [37]. Krzycki et al. reported that L-Lys is the only precursor of L-Pyl. By providing isotopically labeled L-Lys to methanogenic *Archaea* with the *pylTSBCD* gene cluster, methylamine methyltransferase with L-Pyl incorporation was obtained via mass spectrometry analysis and purification. Further, the biosynthetic process of the converting two L-Lys molecules into one L-Pyl molecule was revealed [38]. The *pylBCD* genes are used for L-Pyl synthesis with tRNA-independent [39], while the *pylT* gene can produce tRNA_CUA_ (also called tRNA^Pyl^), and the *pylS* gene can encode pyrrolysyl-tRNA synthase [40]. Further, the introduction of *pylTSBCD* genes into *E. coli* can enable the incorporation of endogenously biosynthesized pyrrolysine into proteins. The L-Pyl production capacity of *E. coli* was improved by Ho et al. via rational engineering and the directed evolution of the whole biosynthetic pathway of L-Pyl. They also developed alternating phage-assisted non-continuous evolution (Alt-PANCE), also known as alternating mutagenesis and selective phage growth, to accommodate the toxicity of L-Pyl biosynthetic genes [41]. The evolutionary pathway enabled a 32-fold increase in pyl-incorporating protein yield compared to the rationally modified pathway. The evolved PylB mutant had a 4.5-fold increase in intracellular levels and a 2.2-fold increase in protease resistance.

Gamma-aminobutyric acid (GABA) with high nutritional value can be produced by lactic acid bacteria [42,43,44]. The GABA production capacity of *Lactiplantibacillus plantarum* was improved by changing the crucial fermentation parameters. The optimization of the inoculum percentage, initial pH, inorganic ions, and nutrients concentration significantly improved the ability of a strain to produce GABA [45].

Selenium is an essential micronutrient that can be incorporated into the active site of specific selenocysteine proteins in the organism through the form of selenocysteine. Selenium-containing proteins play an important role in the regulation of organisms and can be used as research targets for the treatment of some diseases, including cancer, diabetes, Alzheimer’s disease, mental disorders, cardiovascular diseases, etc. [46,47,48]. Normally, biosynthetic ncAAs are formed in the cytoplasmic matrix, which is then linked by aaRS to the corresponding tRNA, thereby completing the incorporation into protein. The biosynthetic pathway of selenocysteine is different from that of ordinary ncAAs. Selenocysteine has a homologous tRNA^Sec^, but there is no free selenocysteine in the cytoplasmic matrix and no corresponding selenocysteinyl-tRNA synthase. The synthesis of selenocysteine does not begin with the ligation of selenocysteine to homologous tRNA^Sec^, but rather the seryl-tRNA synthase first attaches L-Ser to non-homologous tRNA^Sec^ to form seryl-tRNA^Sec^. In bacteria, selenocysteine synthase (SelA) directly acts on seryl-tRNA^Sec^ and removes hydroxyl group from seryl group to generate an intermediate. The intermediate then receives the activated selenophosphate to eventually form selenocysteinyl-tRNA^Sec^. Subsequently, selenocysteinyl-tRNA^Sec^ is paired with UGA codon to complete the incorporation of selenocysteine into the protein [49,50,51].

Genome-scale models (GEMs) of metabolism are a new technology composed of the full inventory of metabolic reactions encoded by the genome of an organism, and they have been used to achieve the efficient production of ncAAs [52]. GEMs can explore trade-offs between the growth rate and production, while computer simulations can be used to analyze metabolic pathways and identify strategies for improving production. For a specific example, the *papBAC* gene cluster from *Pseudomonas fluorescens* was introduced into *E. coli* strain EcNR2 to achieve the production of *p*-amino-phenylalanine (*p*AF), but there was still a trade-off between *p*AF production and the growth rate [53]. To increase *p*AF production, a GEM of *E. coli* metabolism with computer design was used to identify metabolic pathways and determine the recombinant strain metabolism. Upregulating the metabolic flux in the chorismate biosynthetic pathway by eliminating feedback inhibition was the most effective strategy for increasing *p*AF production [54]. This study demonstrates the power of GEMs and computational analysis in terms of optimizing metabolic pathways and improving the production of valuable compounds.

The construction of efficient microbial cell factories for ncAAs has become popular in recent years. These cell factories are mainly created by reconstructing synthesis pathways, designing and modifying key enzymes, coordinating precursor regulation, knocking out competing bypass pathways, constructing cofactor regeneration systems, and intelligently regulating the fermentation process. So far, only a few biosynthetic pathways of ncAAs have been confirmed [11]. Advancements in synthetic and computational biology technologies, as well as multidisciplinary collaborations, have begun to shed light on ncAAs biosynthesis. In the future, the precise design of ncAAs biosynthetic pathways may be accomplished using advanced bioinformatics or biosynthesis simulation tools. Additionally, more chassis with high tolerance to specific ncAAs must be engineered or screened to increase the compatibility between microbes and heterologous ncAAs biosynthetic pathways.

## 3. Methods for ncAAs Incorporation into Proteins

### 3.1. GCE Methods for ncAAs Incorporation into Proteins

The biosynthesis of ncAAs and their incorporation into target proteins within a single microbe has become an attractive application of these molecules. This approach allows the site-specific labeling or modification of proteins with ncAAs, which can improve the properties of proteins and the interactions between peptides. Recent advancements in genetic engineering techniques have enabled the production of the enzymes with the incorporation of ncAAs by replacing cAAs with non-canonical ones at specific sites. In addition, the optimization of the efficiency and selectivity of orthogonal reactions can enable the selective labeling of tailor-made proteins with fluorophores or other probes. The ability to produce and incorporate ncAAs into proteins within the same microbe provides significant advantages over traditional incorporation methods, which are based on chemical synthesis, enzyme conjugation, and in vitro translation. These traditional methods are time-consuming and expensive and often require post-translational modifications (PTMs) to proteins. The biosynthesis of ncAAs and the incorporation into target proteins within the same microbe relies on a technology of genetic code extension (GCE).

**Figure 2:**
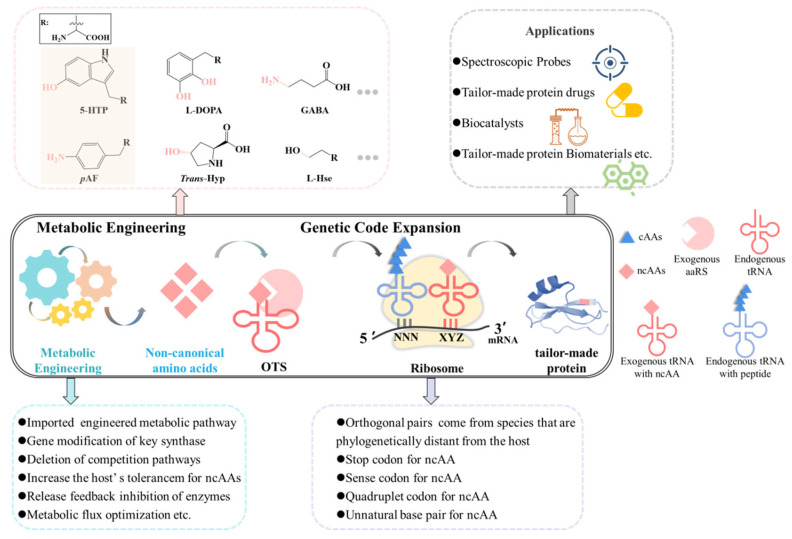
A diagram of the combination of metabolic engineering and GCE. The diagram includes the metabolic biosynthesis of ncAAs, incorporation into proteins via GCE, and applications of tailor-made proteins. 5-HTP [5] and *p*AF [79] have been biosynthesized in vivo, incorporated into proteins, and implemented for applications.

The process of GCE for integrating ncAAs into proteins requires a rigorous and efficient translation mechanism similar to the natural translation process. For GCE to work, each amino acid is matched to its corresponding codon and an orthogonal translation system (OTS). This OTS contains exogenous tRNA and exogenous aminoacyl-tRNA synthetases (aaRS), which follow strict orthogonality criteria. There are two major ways to expand the amino acid repertoire: chemical aminoacylation of tRNA [55,56] and enzyme-mediated (aaRS) aminoacylation of tRNA, which can generate an aminoacyl-tRNA (aa-tRNA) that is active for protein synthesis on the ribosome. The aminoacylated tRNA carries the ncAA, along with the reassigned and desired codons, thereby facilitating the incorporation of the ncAA into the extended peptide chain (Figure 2). It is important that the orthogonal system do not cross-react with the endogenous translation system, as this issue will lead to confusion in the translation system. To prevent false cross-reactions, each orthogonal translation system must undergo multiple rounds of positive and negative screening.

#### 3.1.1. GCE Based on Stop Codon Suppression (SCS)

In the natural translation system, there are three types of stop codons: UAG (amber codon), UAA (ochre codon), and UGA (opal codon). These codons do not encode any amino acids but mediate the termination of the translation process. Release factors (RFs) are proteins that are responsible for recognizing and binding to these stop codons, leading to the release of the synthesized protein. Interestingly, there is some redundancy in the genetic code when it comes to stop codons. For example, RF1 can recognize both UAA and UAG, while RF2 can recognize both UAA and UGA. This difference means that there is at least one ‘redundant’ codon that can potentially be repurposed to encode a different amino acid. In *E. coli*, Schultz et al. chemically acylated a suppressor tRNA to encode a ncAA in response to the ‘sense codons’ position replaced by a stop codon UAG [57]. This method is known as stop codon suppression (SCS), which takes advantage of the degeneracy of the three stop codons and the low abundance of the stop codon UAG (Figure 2, XYZ = Stop codon). Moreover, Pastore et al. used GCE based on SCS to replace a single tryptophan residue in a copper protein amicyanin with ncAA 5-HTP. The incorporation of 5-HTP changed the fluorescence emission maximum of amicyanin from 318 to 331 nm. Moreover, the fluorescence quantum yield of the 5-HTP-containing amicyanin was much less than that of the native amicyanin [58]. This example illustrated that the incorporation of ncAAs changed the physical properties of proteins.

Both RFs and suppressor tRNAs have the ability to recognize stop codons. However, competitive recognition between these factors can result in the premature termination of translation, leading to protein truncation and reducing the incorporation efficiency of ncAAs into protein. To address this issue, Lajoie et al. utilized multiplex automated genome engineering (MAGE) and conjugative assembly genome engineering (CAGE) techniques to replace all 321 UAG codons with synonymous UAA codons in the *E. coli* genome [59]. Additionally, they deleted the *prf*A gene, which encodes RF1 without affecting the strain growth. A genomically recoded organism (GRO) known as *E. coli* C321.ΔA was created. This strain no longer recognized UAG as a stop codon. This strain has higher efficiency for the incorporation of ncAAs compared to the wild-type strain [60,61]. So far, only *E. coli* C321.ΔA was obtained and used as a chassis. It is crucial to develop other chassis with modified genomes to expand the range of applicable hosts for incorporating ncAAs into proteins based on this principle. Overall, SCS allows the introduction of ncAAs into specific positions in a protein, expanding the possibilities of protein engineering and modification.

#### 3.1.2. GCE Based on Synonymous Codon Compression

In total, 18 of the 20 cAAs have two or more codons. L-Leu, L-Arg, and L-Ser are encoded by up to six synonymous codons, while only L-Met and L-Trp are encoded by one codon. Hence, utilizing the degeneracy of codons to replace synonymous codons with a different codon at the genome-wide scale and deleting the tRNAs that decode them can release the replaced codons from the standard genetic code and reassign the replaced codons to ncAAs. This method is called synonymous codon compression [62]. By reducing the number of synonymous codons encoding cAAs, the recoded codons can be freed up for the incorporation of ncAAs or other non-amino acid molecules (Figure 2, XYZ = Sense codon). As an example, Fredens et al. replaced L-Ser codons (UCG, UCA) and a stop codon (UAG) with their synonymous codons (AGC, AGU, UAA) and deleted the corresponding tRNA and the RF1 to construct an *E. coli* strain called Syn61 [63]. This process allowed the incorporation of three ncAAs into a single protein in the engineered strain [64]. However, the reassignment of sense codons at the genome level is still a major technical challenge. Completely eliminating a large number of codons from the genome is not a straightforward task, and the replacement of synonymous codons can have various effects on gene expression, cell fitness [65], and translation speed [66,67]. Additionally, the current approach is primarily limited to bacterial hosts and is not easily applicable to more complex eukaryotic systems.

#### 3.1.3. GCE Based on Other Approaches

Another method of incorporating ncAAs into proteins is to use quadruplet or quintuplet codons, which are called ‘frameshift suppression’. Unlike the redistribution or reassignment of codons, this method allows the expansion of the genetic code without making extensive modifications to the genome. ‘Frameshift suppression’ provides the potential to expand from the 64 codons of the natural genetic code to 4^4^ = 256 [68] or 4^5^ = 1024 [69] ‘blanking’ codons. The use of quadruplet codons provides greater flexibility, as it allows the incorporation of ncAAs without the need for large-scale genome modifications. This method also helps to reduce the potential cross-decoding effects of endogenous triplet codons [70,71,72]. Quadruplet codons have been primarily used in single-celled systems, such as the nematode *Caenorhabditis elegans*. The application range is currently limited, and the efficiency is currently low. Efforts to improve the efficiency of quadruplet codon decoding have focused on directed evolution techniques, including the evolution of anticodon loops of tRNAs and the evolution of the ribosome decoding center in the chassis [73]. The use of quadruplet codons is an interesting and promising method of incorporating ncAAs into proteins. Further work needs to concentrate on expanding its applicability and improving decoding efficiency.

Moreover, unnatural base pairs (UBPs) provide another alternative for expanding the genetic codon library. For example, Bain et al. incorporated the ncAA L-iodotyrosine into proteins using an UBPs (*iso*-C)AG-tRNA_CU (*iso*-dG)_. The (*iso*-C)AG was called the 65th codon [74]. After that, several UBPs were developed [75,76,77]. Malyshev created a synthetic base pair called X-Y, where X represents dNaM and Y represents d5SICS [75]. UBPs involve the introduction of new codon–anticodon interactions, enabling the site-specific incorporation of ncAAs into proteins through ribosomal-mediated translation. In a six-letter unnatural bases system, it is theoretically possible to generate 152 new codons for ncAAs [78]. The use of UBPs allows the expansion of the genetic code in a novel form, providing additional opportunities for the incorporation of ncAAs into proteins. This technology holds promise for the development of new functions and properties of proteins.

### 3.2. Selective Pressure Incorporation (SPI)

**Figure 3:**
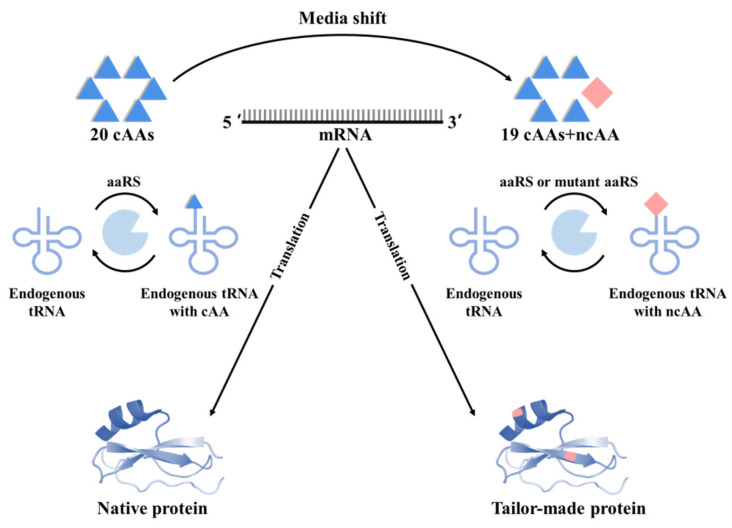
SPI. The incorporation of ncAAs into proteins is realized via an endogenous orthogonal system. Exogenous ncAA analogs are supplemented in the medium to induce the protein translation based on the availability of ncAAs with the assistance of the wild-type or mutated aaRS.

Selective pressure incorporation (SPI), also known as ‘residue-specific incorporation’, is a method that relies on auxotrophic hosts that are deficient in one or more specific cAAs [80]. This approach is based on the substrate tolerance of cellular systems [81]. Endogenous aaRS play a crucial role in replacing the cAAs in protein with one or more amino acid analogs that have similar structures to those of the cAAs [82]. The auxotrophic hosts are cultured in a medium that initially contains a limited amount of nAAs. As the cells grow and begin to express recombinant proteins, the cAAs are gradually depleted. At an appropriate growth state, exogenous ncAA analogs are supplemented in the medium. The expression of target protein gene is regulated by an inducible promoter, ensuring that protein translation is dependent on the availability of ncAAs in the medium [83] (Figure 3). This method is commonly used to modify ribosomal synthetic and post-translationally modified peptides by incorporating ncAAs. Through adding ncAAs in the medium, unique functional polypeptides can be produced. SPI enables the specific modification of residues to create novel functions and chemical diversity in peptides or proteins [84,85,86].

SPI allows the incorporation of multiple ncAAs throughout the proteome. This process can lead to a cumulative or synergistic effect of the ncAAs, resulting in more significant changes in the properties of the target proteins compared to a single substitution. However, SPI is more suitable for ncAAs that have similar properties to those of cAAs. This limitation restricts the application of SPI for ncAAs with distinct or unique properties. Additionally, SPI does not guarantee the specific incorporation of a single site, which is not conducive to studying the fine modification of protein sites or precise control of protein modification. Despite these limitations, SPI remains a valuable tool for introducing ncAAs into proteins to create tailor-made proteins with expanded chemical diversity.

### 3.3. Solid-Phase Peptide Synthesis (SPPS)

**Figure 4:**
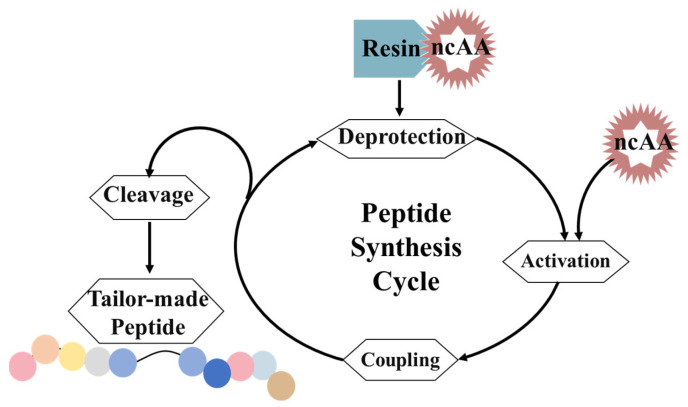
SPPS. In SPPS, the C-terminal of the first amino acid protected by an amino-protecting group needs to connect to the resin via a covalent bond. After that, the protective group is removed to expose a free amino group, which can be coupled with the next amino acid. The general principle of SPPS consists of a repeated cycle of coupling–flushing–deprotection–flushing–coupling.

When an endogenous expression system for producing tailor-made proteins is unavailable or impractical, in vitro methods can be used to incorporate ncAAs into proteins. One commonly used method for in vitro protein synthesis is solid-phase peptide synthesis (SPPS) (Figure 4). SPPS involves the step-by-step assembly of peptide chains attached to the insoluble resin carriers. Multiple amino acids are linked together through peptide bonds to produce peptides. In SPPS, the C-terminal of the first amino acid, which is protected by an amino-protecting group, is connected to the resin via a covalent bond. The protective group is then removed to expose a free amino group, which can be coupled with the next amino acid. This process is repeated, extending the length of the peptide chain. The general principle of SPPS involves a repeated cycle of coupling–flushing–deprotection–flushing–coupling. The polypeptide remains immobilized on the solid phase throughout the synthesis process. After assembly, the polypeptide is separated from the resin using trifluoroacetic acid, and the protective group is removed. Commonly used amino-protecting groups include 9-fluorenylmethoxycarbonyl (Fmoc) and *t*-butyloxycarbonyl (*t*Boc) [87,88]. SPPS has practical applications in the chemical synthesis of recombinant proteins using ncAAs [87,89,90]. It can be used to synthesize the peptides that are difficult to endogenously express, such as those composed of D-amino acids. SPPS is also useful for modifying the main chain of peptides or proteins. It is particularly suitable for incorporating amino acid analogs that may be toxic to cells or incompatible with cellular translation mechanisms. The chemical synthesis of peptides is limited by the length of peptide (<50~60 amino acids) [91,92] and the speed of synthesis. Overall, SPPS is a valuable tool for incorporating ncAAs into proteins in vitro, allowing the synthesis of peptides and proteins that are challenging or impossible to produce using traditional endogenous expression systems.

In addition, the split inteins or sortases have been applied for protein ligation [93,94] to enable the incorporation of unnatural peptides and non-peptide molecules into proteins, which is beneficial to realize large-scale protein modification using SPPPS technology. Split inteins can link two explicit peptides into a new protein through the trans-linking of proteins. Sortases found in Gram-positive bacteria have the function of linking proteins [95]. Boder et al. described the sortases as a molecular ‘stapler’ [96]. They covalently linked the labeled model protein (green fluorescent protein) to chemically modified polystyrene beads using the sortase A from *Staphylococcus aureus*.

### 3.4. Cell-Free Protein Synthesis (CFPS)

**Figure 5:**
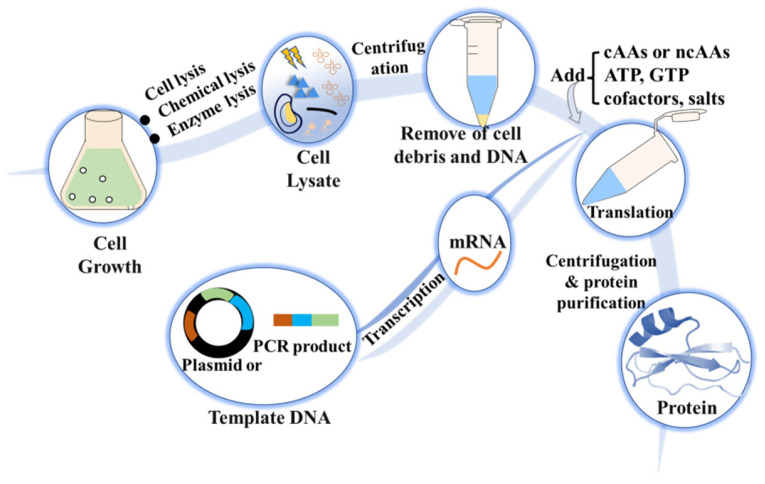
CFPS. Mixing exogenous DNA or mRNA, cell lysate, ncAAs, ATP, and cofactors salts could achieve the synthesis of tailor-made proteins via ncAAs incorporation.

Cell-free protein synthesis (CFPS) is another in vitro method used to synthesize tailor-made proteins. CFPS is a rapid and high-throughput expression technique that allows the production of proteins using exogenous DNA or mRNA as a template [97,98] (Figure 5). This method requires the use of multiple enzymes provided by cell lysates to supply substrate and energy. CFPS systems are available based on different cell lysates, including *E. coli* lysate, yeast extract, rabbit reticulocyte lysate, or wheat germ extract [99,100,101]. Each system offers unique advantages and may be preferred depending on the specific requirements of protein synthesis. The in vitro CFPS systems allow flexibility and freedom in protein design, making them powerful tools for protein engineering and biopharmaceutical production. CFPS has been successfully scaled up for manufacturing purposes. However, CFPS also have some limitations. For instance, the reaction duration is relatively short, which can result in low protein yields. The cost of CFPS is high due to the need for specialized reagents and enzymes. Despite these limitations, CFPS remains a valuable tool for the rapid synthesis of tailor-made proteins in vitro, offering advantages such as flexibility and high-throughput capability. Currently, there are many studies that continue to address the limitations of CFPS and improve its efficiency and scalability for wider application.

### 3.5. Other Methods for the Incorporation of ncAAs

In addition to the above methods, post-translational mutagenesis and cysteine functionalization are the preferred methods of incorporating ncAAs into proteins. Natural PTMs form bonds with heteroatoms (non-carbon) at the γ (CysSγ, ThrOγ, SerOγ) or ω (LysNω, TyrOω) position of the side chain, while post-translational mutagenesis uses free radical chemistry to form carbon–carbon bonds between amino acid residues and selected functional groups, which can introduce functions or labels into a wide range of proteins [102]. Unpaired free cysteine often exhibits high nucleophilicity and is relatively rare among proteins, making it an ideal site for protein modifications. Cysteine residues can be easily introduced into proteins via site-specific mutagenesis [103]. Cysteine-specific modification is usually achieved via the reaction of sulfhydryl groups with electrophilic reagents, such as iodoacetamides, pyridine disulfide, maleimides, and alkyl halides [104]. Cysteine modification is a practical method used to produce functional proteins with a wide range of biomedical applications.

## 4. The Applications of Tailor-Made Proteins

### 4.1. Tailor-Made Protein Materials

The incorporation of ncAAs into proteins provides unique chemical and biological functions for new materials with extraordinary properties and functions. Compared to chemical crosslinking, photo-crosslinking can produce new biomaterials under milder conditions. Conticello et al. introduced the ncAAs *p*-benzoyl-L-phenylalanine (Bpa) and *p*-azido-phenylalanine (AzF) into elastin mimetic polymers, which enhanced the protein function [105]. Montclare et al. studied the effects of fluorination on fiber assembly, supramolecular assembly, and mechanical properties [106]. They achieved the incorporation of trifluoroleucine (TFL) into α-helical coiled-coils C and Q through residue-specific incorporation. Compared to the non-fluorinated variants, the generated C-TFL and Q-TFL had increased α-helix, enhanced drug binding capacity, improved thermal stability, and enhanced fiber assembly in the alkaline environment. Due to the overproduction of special mussel adhesion proteins, marine mussels are able to firmly adhere to various wet surfaces. One of the key amino acids involved in special mussel foot proteins is L-3,4-dihydroxyphenylalanine (DOPA) containing a catechol group [107,108,109]. The adhesive property of these proteins relies on the post-translational hydroxylation of L-DOPA. This modification can only be completed in eukaryotic cells. Therefore, the recombinant production of these proteins in prokaryotic cells remains a significant challenge. Overcoming this challenge would have important implications for the engineering and design of new adhesives and coatings with improved underwater performance [110]. One approach used to address this challenge is to develop efficient orthogonal aaRS/tRNA pairs. Using the orthogonal aaRS/tRNA pairs to incorporate the photoactivatable ncAAs (*O*-nitrobenzyl DOPA) into proteins can achieve the production of mussel adhesion proteins in vivo [111]. The incorporation of DOPA-like ncAAs into proteins using an orthogonal system offers the possibility of developing bio-adhesives and coatings that mimic the adhesive properties of marine mussels.

### 4.2. Tailor-Made Protein Probes

Proteomics presents challenges in analyzing protein–protein interactions within cells. Current methods often rely on affinity purification, but it is difficult to isolate and purify the complete protein complexes from cells. This issue can result in incomplete protein–protein interactions, as some proteins may dissociate during cell lysis or affinity purification. Alternative methods that utilize fluorescent protein photo-crosslinking probes to target proteins of interest (POIs) have been developed [112,113,114,115,116]. These probes have become valuable tools for studying intracellular protein–protein interactions. By incorporating fluorescent ncAAs into proteins through site-specific incorporation, micro-organisms can be modified to allow live imaging and tracking [117]. For example, Curnew et al. utilized an orthogonal tRNA/synthetase pair to incorporate the fluorescent ncAA Anap (3-[(6-acetyl-2-naphthalenyl)amino]-L-alanine) into the core protein of hepatitis C virus (HCV) to generate a visible HCV. Anap is a derivative of 6-propionyl-2-(*N*,*N*-dimethyl)-aminonaphthalene (prodan), which has an environmentally sensitive fluorophore [117]. The fluorescent ncAA labeling method can eliminate the need for antibodies or labels to achieve protein visualization [118]. Moreover, special reactions with the ucAAs in the protein can visualize the protein. For example, Praveschotinunt et al. incorporated *p*-azido-L-phenylalanine (*p*AzF) into *E. coli* in response to the UAG stop codon. When *p*AzF was incorporated into CsgA, the strain could be covalently labeled with a Cy5 dye using a dibenzocyclooctyl (DBCO) group through a copper-free orthogonal reaction [49]. Fluorescent protein probes have also become powerful tools for exploring protein conformational changes, localization, and molecular interactions [119]. These probes have fluorescent properties that offer several advantages over traditional methods, including small size, ease of use, an ability to provide various colors, and improved photochemical properties. The synthesis of fluorescent probes provides numerous benefits for the in-depth study of protein properties and interactions.

### 4.3. Tailor-Made Protein Drugs

The development of modern drugs mainly focuses on the screening of chemical compounds, with limited emphasis on the design and modification of protein drugs. So far, the tailor-made protein drugs have emerged. The special functional groups of ncAAs have greatly expanded the design space for protein drugs, bringing new developments to the biomedical field [120,121]. One strategy for developing covalent protein drugs is based on proximity-enabled reactivity (PERx) [122,123]. This approach needs to introduce an aromatic fluorosulfate group into the side chain of tyrosine and lysine residues in proteins, such as human programmed cell death protein-1 (PD-1). The ncAAs FSY (fluorosulfate-L-tyrosine) and FSK (fluorosulfonyloxy-benzoyl-L-lysine) are designed based on sulfur–fluorine replacement reactions with proximate lysine, histidine, and tyrosine residues. The covalent modification of PD-1 with FSY and FSK can result in stronger anti-tumor effects compared to wild-type PD-1 in immunized mice. This strategy highlights the potential of ncAAs in terms of developing covalent protein drugs with enhanced therapeutic efficacy. Another important development in targeted anticancer drugs is the use of antibody–drug conjugates (ADCs) [124,125,126]. ADCs involve the site-specific conjugation of ncAAs, providing a new approach that overcomes the limitations of conventional modifications dependent on cysteine residues [127]. This site-specific conjugation of ncAAs allows ADCs to exhibit improved pharmacokinetics and antigen binding properties and higher titers. These advancements expand the possibilities of the development of targeted therapies for cancer.

Interleukin-2 (IL-2), as a promising therapeutic agent for autoimmune diseases, is a cytokine that can selectively bind to the trimer IL-2 receptor (IL-2R) on regulatory T (Treg) cells and preferentially activate Treg cells. However, the therapeutic window of IL-2 is narrow, and the half-life is short. Zhang et al. proved that the pharmacokinetics and half-life of IL-2 can be significantly improved via the orthogonal conjugation of cytokines with polyethylene glycol (PEG) moieties by adding azide-containing amino acids at certain sites via copper-free click reaction [128]. Site-specific PEGylation can be widely used to engineer cytokines in order to improve the therapeutic performance of autoimmune diseases.

The CRISPR-Cas system is widely used in the genome editing and gene regulation of human cells and other biological cells [129]. The DNA cleavage complex of the systems are composed of a Cas nuclease and a CRISPR RNA(crRNA). Compared to Cas9 nuclease derived from *Streptococcus pyogenes*, the Cas12a (Cpf1) has a lower incidence of off-target effect, enabling Cas12a to be a potentially safe choice for clinical application. However, due to its relatively low efficiency in editing human genome, CRISPR-Cas12a system has not been widely used. Ling et al. proposed that the low editing efficiency of Cas12a may be due to the relatively weak interaction between the Cas protein and the crRNA [130]. They site-specifically modified the Cas12a via GCE with an azide-containing ncAA 4-(2-azidoethoxy)-l-phenylalanine (AeF). The result showed that the modified system had higher editing efficiency compared to that of wild type CRISPR-Cas12a.

Overall, the incorporation of ncAAs into protein drugs offers new avenues for drug design and modification, enabling the development of covalent protein drugs with enhanced therapeutic effects and the creation of more effective targeted therapies, such as ADCs.

### 4.4. Applications of ncAAs in Vaccines

GCE technology offers a groundbreaking solution for the development of therapeutic and prophylactic vaccines. For example, PTMs of L-tyrosine residues in proteins lead to the formation of nitrotyrosine and sulfonyl tyrosine, which have been found to be associated with the breaking of immune tolerance and the induction of autoimmune diseases. The nitro and sulfonyl groups possess strong immunogenicity, which is crucial for breaking self-immune tolerance [131]. Moreover, Grünewald et al. utilized GCE to mutate the 11th L-Lys or the 86th L-Tyr residue in murine tumor necrosis factor-alpha (mTNF-α) into pNO_2_Phe [132]. These modifications introduced a new antigenic determinant in mTNF-α, effectively breaking autoimmune tolerance and generating high titers of antibodies that could recognize both the mutant and wild-type mTNF-α [133,134]. This result demonstrates the potential of GCE in terms of developing vaccines that target specific PTMs and break immune tolerance. GCE technology has also been applied in the development of preventive attenuated vaccines. For example, Zhou et al. randomly selected a codon on NP protein in influenza virus and mutated the codon into amber codon UAG. The mutated virus gene was then packaged by transgenic human embryonic kidney (HEK) 293T cells containing the *Methanosarcina barkeri* MS pyrrolysyl-tRNA synthetase/tRNA_CUA_ (*Mb*PylRS/tRNA_CUA_) pair. Only in the presence of 1 mM ncAA N^ε^-2-azidoxycarbonyl-L-lysine (NAEK) did transgenic cells show cytopathic effect (CPE) [135]. The replication-incompetent influenza virus with a low escape rate can be directly transformed to vaccines, retaining the complete structure and infectivity of the virus, thereby transforming the influenza virus from a lethal source of infection into a preventive vaccine. This defective attenuated vaccine demonstrated stronger immunogenicity compared to commercial vaccines, eliciting a more robust and extensive immune response in the host immune system. As an example, Ji et al. used the above method to transform influenza A virus (IAV) into a tumor treatment vaccine and verified it using a mouse tumor model [136]. They developed a chimeric antigenic peptide influenza virus system-CAP-Flu, which was used to deliver the antigenic peptide combined with influenza A virus (IAV) to the lungs via inhalation, thereby treating lung cancer and preventing the metastasis of various tumors to the lungs. The engineered IAV can be combined with any new tumor antigen of interest to produce a new therapeutic vaccine for lung cancer. These examples highlight the versatility and potential of ncAAs in vaccine development. GCE technology allows the precise modification of proteins to generate novel antigenic determinants, enhancing immunogenicity and leading to effective vaccines. The broad application of ncAAs in vaccines holds promise for improving preventive and therapeutic strategies against various diseases.

## 5. Perspectives

Although ncAAs have exciting potential, there are still some challenges in terms of their application and implementation. One challenge is the lack of clear biosynthesis pathways for some ncAAs and the limited knowledge of critical enzymes in these pathways. Enzymes are crucial for constructing microbial cell factories of ncAAs. Currently, the properties, structure, and interactions between enzymes and ncAA biosynthesis pathways or microbes are poorly understood [137]. Therefore, the simulation prediction of enzyme structures and biosynthesis pathways is needed to reveal and elucidate the metabolic pathways and key enzymes of ncAAs biosynthesis. Understanding these pathways will help to combine the production and incorporation of ncAAs to generate the desired proteins. Another challenge is the development of a diverse set of codon-aaRS-tRNA orthogonal systems that exhibit sufficient efficiency and reading fidelity. The orthogonal systems are crucial for expanding the application of ncAAs. The design and modification of orthogonal systems are essential for achieving the precise and reliable incorporation of ncAAs into proteins. The multiple strategies of GCE have challenged the ‘frozen accident hypothesis’ [138] of the genetic codon and demonstrated the plasticity and evolvability of the standard genetic code. This observation creates new possibilities for the design of the advanced life forms and the incorporation of ncAAs.

Due to the paucity of ncAAs biosynthetic pathways [139] and the frequent incompatibilities between GCE techniques and pathways [140], only a few ncAAs [5,79,141] can be biosynthesized and incorporated into proteins in vivo. By leveraging synthetic biology and evolutionary engineering, it is anticipated that more pathways for ncAAs biosynthesis can be constructed in suitable chassis in the future. This development will enable the biosynthesis and incorporation of desired ncAAs into target protein within the same microbe. We can expect the discovery of more ncAAs, biosynthetic pathways, physiochemical properties, and structures related to tailor-made proteins. Generally, addressing the challenges associated with ncAAs will require interdisciplinary efforts involving biochemistry, molecular biology, synthetic biology, and systems biology.

## Data Availability

Not applicable.

## Sample Availability

Samples of the compounds are unavailable from the authors.
